# End-to-End Intelligent Drug Discovery via a Scalable and Explainable Graph-Transformer Framework

**DOI:** 10.3390/bioengineering13090961

**Published:** 2026-08-23

**Authors:** Fatma M. Talaat, Ahmed Elnakib, Asmaa A. Hekal, Mona Alnaggar, Ahmed Gamal Abdellatif, Mahmoud A. Shawky, Soha Safwat, Warda M. Shaban, Mohamed Shehata

**Affiliations:** 1 Faculty of Artificial Intelligence, Kafrelsheikh University, Kafrelsheikh 33516, Egypt; fatma.nada@ai.kfs.edu.eg; 2 School of Engineering, Penn State Erie-The Behrend College, Erie, PA 16563, USA; 3 Faculty of Computers and Information Systems, Egyptian Chinese University (ECU), Cairo 11765, Egypt; asmaa.aboulfadl@ecu.edu.eg (A.A.H.); ahmed.gamal@ecu.edu.eg (A.G.A.); soha.safwat@ecu.edu.eg (S.S.); 4 Department of Robotics and Intelligent Machines, Faculty of Artificial Intelligence, Kafrelsheikh University, Kafrelsheikh 33516, Egypt; mona.alnaggar@ai.kfs.edu.eg; 5 School of Computer Science and Electronic Engineering, University of Essex, Colchester, CO4 3SQ, UK; mahmoud.a.shawky-ahmed@essex.ac.uk; 6 The Egyptian Technical Research and Development Center, Cairo 11618, Egypt; 7 Faculty of Artificial Intelligence and Informatics, Horus University-Egypt, New Damietta 34517, Egypt; wmohammed@horus.edu.eg; 8 Computer Science Program, James C. Bowling School of Business, Midway University, Midway, KY 40347, USA

**Keywords:** drug discovery, explainable artificial intelligence (XAI), graph neural networks, graph transformer, molecular representation

## Abstract

Drug discovery is still an expensive and time-consuming process where finding the right drug associations is important for therapeutic development. In this paper, a new system is proposed for drug design called PharmaGraphFormer (PGF). It consists of five stages: (i) Data acquisition and preprocessing (DAP), (ii) Feature extraction and feature fusion (FEF), (iii) Molecular representation (MR), (iv) Multi-task prediction, and (v) Explainable artificial intelligence (XAI). This study employs a hybrid graph neural network (GNN)-transformer architecture that combines structural and sequence-based representations. Through DAP, several processes are executed, including the imputation or removal of missing values, outlier rejection, and class balancing. Next, through FEF1, features are extracted to represent the input data efficiently. Initially, compound-protein features are generated to document the interactions and relationships between chemical compounds and their corresponding target proteins. Secondly, drug characterizations are computed to encapsulate the physical, chemical, and structural attributes of each drug. After that, MR is performed using a graph-based molecule representation. Then, a novel model integrating GNNs and graph transformers, termed GNN-T, is proposed. Initially, GNNs represent the most promising deep learning models adept at processing non-Euclidean data. The Graph Transformer layer enhances atom representations by consolidating the representations of adjacent atoms through an attention mechanism. Finally, XAI is applied to explain the internal mechanisms of AI systems, rendering them comprehensible and interpretable. Across five independent runs, the proposed model achieved an accuracy of 0.963±0.002, a precision of 0.971±0.002, a recall of 0.958±0.003, an F1-score of 0.964±0.002, and a ROC-AUC of 0.993±0.001. These results demonstrate an outstanding performance when compared with all other models and emphasize that the proposed model is reliable in solving the problems of prioritizing compounds in line with the latest developments in AI-powered virtual screening and drug–target interaction modeling.

## 1. Introduction

**Figure 1:**
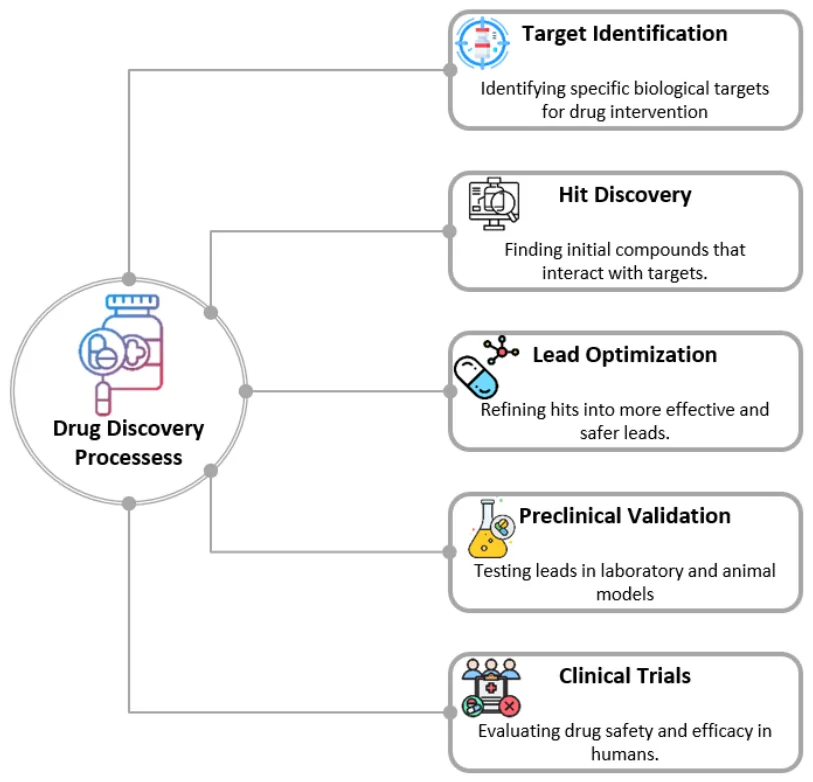
AI in drug discovery and development.

To fight against chronic diseases and infections in a fast and efficient way, there is an absolute necessity for drug discovery processes to become faster and more efficient. This has promoted research into better drug development and the optimization of drug therapies. The process of drug discovery is one of the most costly, lengthy, and difficult processes in biomedical research. A single drug may take more than a decade to develop and cost billions of dollars [1,2]. The overall schematic of a traditional drug discovery process is shown in Figure 1, and involves target identification, hit discovery, lead optimization, preclinical validation, and clinical trials. Although significant advances have been made in drug development technologies, its success rate remains low. Only a small number of drug candidates receive regulatory approval [3]. Most failures have been associated with low efficacy, poor pharmacokinetics, toxicity, or poor target engagement.

In recent years, artificial intelligence (AI) and machine learning (ML) have been proposed to overcome these shortcomings by improving predictive accuracy, reducing experimental costs, and speeding up decision-making throughout the drug discovery pipeline [4]. AI-based approaches have demonstrated strong capabilities in target identification, virtual screening, molecular optimization, drug–target interaction (DTI) prediction, patient stratification, clinical trial design, and drug repurposing. In particular, deep learning methods have had a significant impact on extracting meaningful patterns in large and diverse biological, chemical, genomic, proteomic, transcriptomic, and clinical data. Graph neural networks (GNNs) have become one of the modern AI approaches. Therefore, they are especially significant in computational drug discovery. A graphical representation is a natural representation of molecules; the nodes are atoms, and the corresponding bonds are represented with edges between them.

GNNs have emerged as a pivotal tool for computational drug discovery, especially as molecules can be naturally expressed as graphs, with atoms being graph nodes and chemical bonds being graph edges. This representation enables the model to capture local topological and chemical features relevant to molecular activity and binding behavior. At the same time, transformer-based architectures have demonstrated remarkable success in modeling sequential and contextual dependencies in biological data, especially protein sequences, through self-attention mechanisms that can capture long-range interactions more effectively than conventional recurrent or convolutional approaches.

Recent studies have increasingly explored the use of GNNs for molecular property prediction and DTI modeling, while transformer models have shown strong performance in protein representation learning and sequence-based interaction prediction [5,6]. However, models that rely exclusively on graph-based molecular encoding may fail to adequately capture long-range dependencies in protein sequences, whereas purely transformer-based sequence models may overlook important structural and topological properties of drug molecules. As a result, recent literature has highlighted the need for integrated architectures that combine structural molecular information with sequence-aware biological representations in a unified learning framework. Although hybrid deep learning strategies have shown promise, several important challenges remain insufficiently addressed in the current literature. First, many existing models focus on a single prediction objective, such as binding affinity, without leveraging related biochemical tasks that could improve representation learning and model generalization. Second, model interpretability remains limited. This reduces trust in the biological plausibility of model predictions and makes it hard to adopt in a practical way in pharmaceutical settings. Third, large molecular graphs and high-dimensional biological data still present scalability challenges. Lastly, there are still several challenges in the field concerning dataset bias, incomplete benchmarking, restricted reproducibility, and the absence of standardized validation procedures for regulatory and clinical applications [7,8,9].

These issues point to the fact that, despite rapid methodological advances, the gap between good and predictive models and powerful, interpretable, and deployable AI-based drug discovery is still very wide. In this respect, recent advances in multi-modal and explainable AI suggest that a framework based on the complementary representation of data and open decision-support mechanisms could be more effective for computational drug discovery. This paper proposes a hybrid graph neural network-transformer architecture for complex drug–target modeling. The suggested framework combines sequence-based transformer representations of a target protein with structural graph-based representations of a drug compound, such that both a local molecular representation and a global representation of a molecular target and a drug candidate are obtained. In addition, the framework is made to support multi-task learning to model collectively binding affinity, activity classification, and ADMET-related properties (Absorption, Distribution, Metabolism, Excretion, and Toxicity) in connection with common molecular representations. In order to enhance the transparency of the model, SHapley Additive exPlanations attribution and attention visualization methods are integrated. The framework is tested and trained on a large-scale dataset of drug and target binding affinities, which is evaluated using predictive performance and virtual screening utility. The proposed method offers a practical computational drug discovery framework. In general, the major contributions of this work are summarized as follows:

1. A unified deep learning framework that integrates graph neural networks with transformer-based attention mechanisms to jointly model molecular topology and long-range protein dependencies in drug–target interactions.
2. A multi-task optimization strategy that simultaneously predicts binding affinity, activity class, and ADMET-related properties through shared molecular representations.
3. The incorporation of SHAP-based feature attribution and attention visualization techniques to enhance interpretability and align model predictions with established pharmacophore and medicinal chemistry principles.
4. An efficient computational design based on graph sampling and attention sparsification to improve scalability for large molecular graphs and high-dimensional biochemical datasets.
5. Support for virtual screening, drug repurposing, and molecular design within a single framework.

In contrast to existing hybrid architectures that integrate sequence and graph representations in an ad-hoc manner, PharmaGraphFormer introduces three distinct structural advancements: (1) a dedicated multi-modal feature extraction and fusion (FEF) module that explicitly constructs relational compound-protein interaction descriptors prior to graph representation, (2) an embedded multi-task optimization pipeline jointly addressing continuous binding affinity regression and discrete activity classification, and (3) an intrinsically integrated SHAP-based interpretability mechanism aligned with domain chemistry.

The rest of this paper is organized as follows. Section 2 reviews the related literature, detailing the progress of drug discovery using AI from the well-established computer-aided drug design (CADD) methods to recent hybrid systems. Section 3 explains the process of the proposed framework. The experimental setup, benchmark datasets, and analysis of the results, including performance measures and interpretability case studies, are detailed in Section 4. Finally, Section 5 concludes this work.

## 2. Related Work

AI has become an increasingly important component of modern drug discovery, and it is extensively employed to speed up molecular screening, predict drug–target interactions, estimate pharmacokinetics and optimize toxicity profiles, and support lead optimization [1,10,11,12]. Compared with the classical trial-and-error processes used, AI-based solutions can integrate chemical and biological data that are heterogeneous and enhance the efficiency and accuracy of drug discovery workflows [4,11]. Initial computational methods of drug discovery were based on CADD and quantitative structure-activity relationship (QSAR) models and classical ML algorithms such as support vector machines and random forests [13,14,15,16]. These methods established the foundation for predicting compound activity based on molecular properties. However, their performance is largely dependent on handcrafted features, relying heavily on molecular descriptors and manual feature engineering, which limits their ability to effectively model complex, nonlinear, and high-dimensional biochemical data [11,12].

Recent developments in deep learning have significantly advanced AI-driven drug discovery [17,18,19]. GNNs are one of the methods that have become appropriate in the modeling of molecules. Since molecules can be described naturally in the form of a graph, atoms are represented as nodes; chemical bonds are elements of the edge set. Architectures, including graph attention networks, graph convolutional networks (GCNs), neighborhood aggregation models (GATs), and models of neighborhood aggregation, have demonstrated strong performance in molecular property prediction and drug–target interaction modeling [20,21,22]. These models learn directly from graph structures rather than relying on handcrafted descriptors or learning through trial-and-error, enabling them to more effectively capture local chemical environments and relational dependencies compared with traditional descriptor-based approaches. However, traditional GNNs still have challenges in modeling long-range interactions and capturing global molecular context, particularly in complex biochemical prediction tasks [23,24].

Transformer-based architectures have also attracted increasing attention in drug discovery because of their ability to model contextual relationships through self-attention. Originally developed for natural language processing, transformers have been successfully adapted to molecular and protein sequence representation learning. Models such as Mol-BERT and protein language-based architectures have shown promising results in molecular property prediction and drug–target interaction tasks [25,26,27]. In addition, graph transformer models have demonstrated that attention mechanisms can improve the modeling of long-range dependencies in graph-structured data [24,28]. However, transformer-only approaches may not preserve local topological information as effectively as graph-based methods when molecular connectivity is critical. These observations have motivated the development of hybrid graph-transformer frameworks that combine the complementary strengths of both paradigms. In such systems, graph neural networks are used to capture local structural patterns, whereas transformer layers provide global contextualization and attention over longer-range interactions [27,28]. Such hybrid approaches are particularly attractive for drug discovery tasks that involve both structural and sequence-level information, including compound-protein interaction modeling and related predictive tasks.

A comparison of representative AI-based drug discovery approaches and their relationship to the proposed PGF framework is provided in Table 1.

Another important trend in the recent literature is the use of benchmark datasets and standardized evaluation protocols. Public resources such as LIT-PCBA, MF-PCBA, DOCKSTRING, QMugs, ESOL, and FreeSolv have enhanced transparency and comparability in drug discovery studies [29,30,31,32,33,34]. Nevertheless, variations in dataset composition and task formulations, particularly in definitions, preprocessing strategies, and evaluation settings, make model performance difficult to compare. This is especially the case when evaluating the extrapolation and robustness of high-capacity models like GNNs and transformers. Interpretability is also playing an important role in biomedical AI. Although deep neural architectures achieve strong predictive performance, their inherent black-box nature can be a barrier to scientific trust and regulatory acceptance of them, and its general adoption. The recent literature has highlighted explainable artificial intelligence (XAI) and transparent validation as critical elements of credible AI-based drug development [35,36]. Despite this progress, most of the earlier works continue to focus on predictive performance but do not adequately address the interpretability of the modeling pipeline.

In biomedical computational pipelines, multi-view feature combination has historically relied on techniques such as multiple kernel learning (MKL), which constructs individual kernel matrices for separate biological or chemical descriptor spaces. While MKL combines data sources linearly or non-linearly at the decision boundary, hybrid graph-transformer models operate at the latent representation level. By learning end-to-end representations, GNN-T continuously updates spatial structural relations alongside sequence dependencies rather than relying on static pre-computed distance matrices.

Recent literature indicates significant advancements in graph-based learning and transformer-based models for molecular modeling and drug–target interaction prediction [11,20,27]. However, there are still some significant gaps. The available literature tends to treat the preprocessing, representation learning, prediction, and interpretability as individual aspects as components of an integrated framework. In addition, there are numerous methods based on either graph-based or sequence-based representation, and few studies integrate feature fusion, hybrid graph learning predictors, explainable analysis, and within a unified end-to-end framework. These limitations motivate the present study, which proposes the PharmaGraphFormer, a unified framework that integrates structured molecular representation learning with transformer-based enhancement, predictive modeling, and explainable AI for drug discovery applications. Table 1 summarizes representative AI-based approaches in drug discovery and highlights their relation to the proposed PGF framework. Prior studies have typically focused on isolated components of the pipeline, such as graph-based prediction, sequence-based modeling, benchmarking, or post hoc interpretability. In contrast, PGF is designed to integrate these components within a more cohesive architecture.

**Table 1:** Comparison of representative AI-based approaches in drug discovery and their relation to the proposed Pharma Graph Former (PGF) framework.

Ref	Method	Input	Task	Strength	Limitation	Relation to PGF
[20]	SMGCN	Graph structure with similarity fusion	Drug–target interaction prediction	Effectively exploits structural similarity information	Focuses primarily on DTI tasks with limited interpretability	PGF extends this by incorporating explainability and multi-modal feature integration
[25]	Mol-BERT	SMILES and sequence representations	Molecular property prediction	Captures rich contextual dependencies via attention mechanisms	Lacks explicit encoding of molecular topology	PGF integrates sequence modeling with graph-based structural representations
[26]	Protein language model	Protein embeddings with drug-aware attention	Drug–target interaction prediction	Strong sequence-aware interaction modeling	Limited utilization of molecular graph structure	PGF combines graph learning with transformer-based contextual modeling
[28]	Graph Transformer	Graph-based attention representations	Graph representation learning	Captures long-range dependencies effectively	May overlook fine-grained local structural details	PGF enhances transformer-based graph learning while preserving local structure
[32,33]	Benchmark datasets	Bioactivity datasets	Model evaluation and benchmarking	Enables reproducible and standardized evaluation	Cross-study comparisons remain challenging due to varying protocols	PGF is evaluated within standardized benchmark settings
[35,36]	Explainable AI frameworks	Post-hoc and intrinsic explainability methods	Trustworthy biomedical AI	Enhances model transparency and interpretability	Often applied post hoc rather than integrated into model design	PGF incorporates explainability as an integral component

## 3. The Proposed Pharma Graph Former

**Figure 2:**
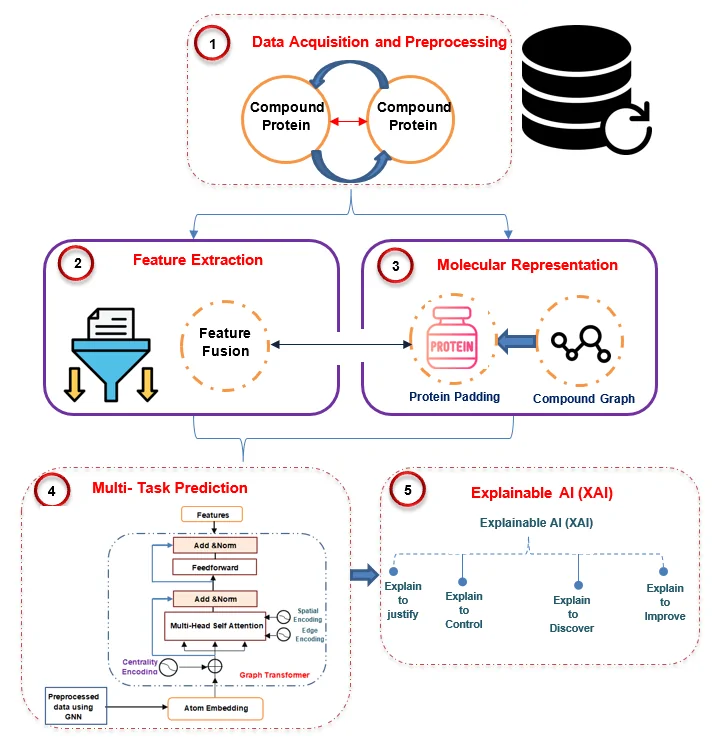
Overview of the proposed pharma graph former framework.

Recent advances in AI-driven drug discovery have shown that accurate molecular prediction increasingly depends on integrating robust preprocessing, informative molecular representation, multi-modal feature learning, and interpretable prediction pipelines [11,20,25,27]. Motivated by these developments, this paper proposes a new framework for AI-driven drug discovery, termed PGF. As shown in Figure 2, PGF consists of five stages: (i) Data acquisition and preprocessing (DAP), (ii) Feature extraction and fusion (FEF), (iii) Molecular representation (MR), (iv) Multi-task prediction, and (v) Explainable AI. The framework is designed to address several limitations in prior work, including fragmented preprocessing strategies, isolated graph-only or sequence-only modeling, and limited integration of interpretability into end-to-end drug discovery systems. The details of each stage are presented in the following subsections.

### 3.1. Data Acquisition and Preprocessing (DAP)

The input data consist of drug-related descriptors, compound-protein interaction information, and molecular structure representations derived from the utilized benchmark dataset(s). These heterogeneous data include both structural and interaction-level information, which motivates the need for preprocessing before downstream feature learning. Accordingly, the data acquisition and preprocessing constitute the first stage of the proposed PGF framework.

**Figure 3:**
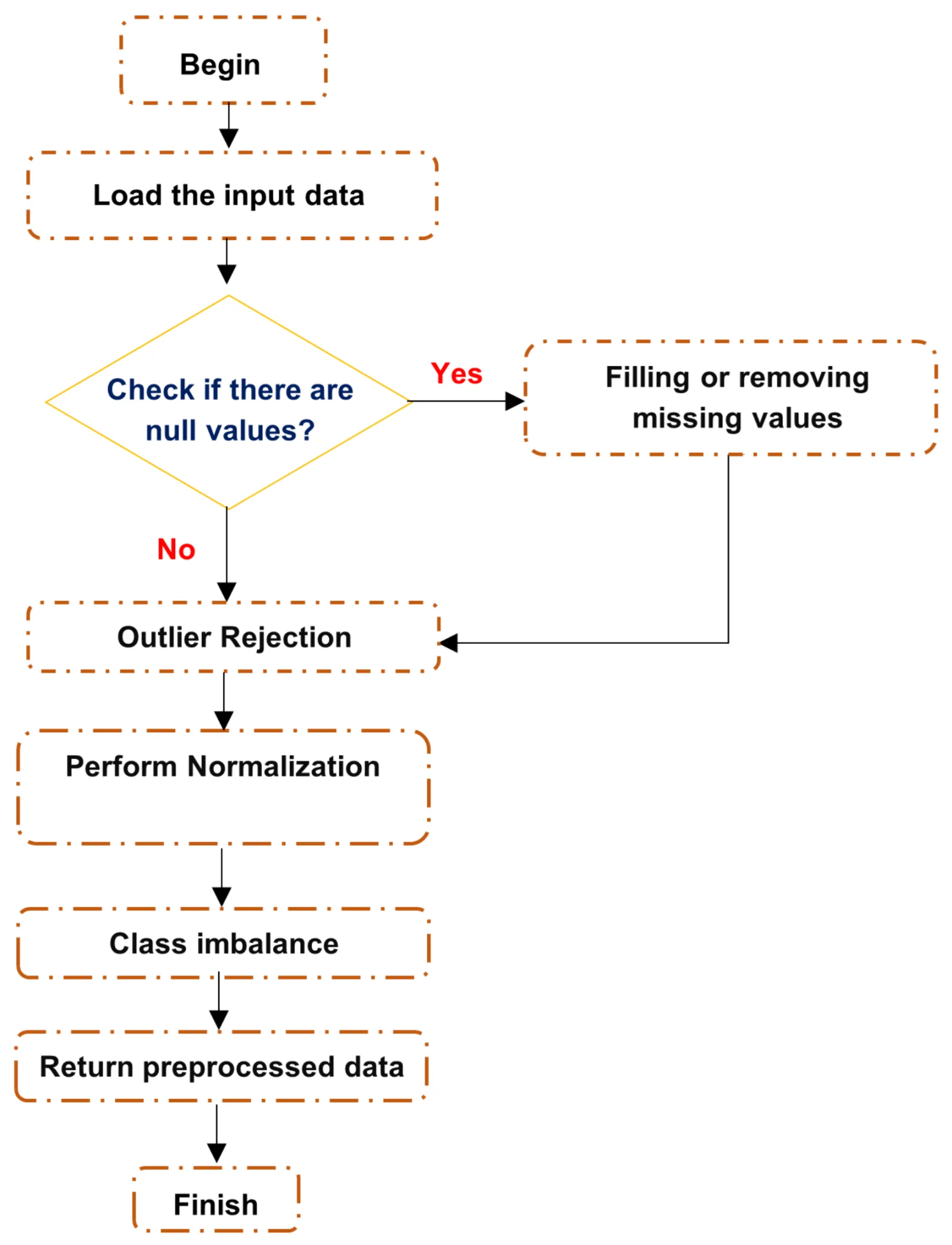
Data acquisition and preprocessing workflow.

This stage prepares the raw data for downstream modeling by addressing common data-quality issues that frequently affect AI-based drug discovery tasks, including missing values, outliers, scale heterogeneity, and class imbalance. These preprocessing measures are necessary since there are differences in the quality of data, and distribution-based feature selection can have a significant impact on predictive model robustness, predictive model reproducibility, and predictive model generalizability, especially in molecular learning benchmarking systems [31,32,33]. As illustrated in Figure 3, the DAP phase involves the processing of missing values, outliers, and their removal, normalization, and adjusting the balance of classes.

Initially, missing values in the input dataset are identified and then either removed or imputed, depending on the extent and nature of the incompleteness. This step is important because incomplete molecular or interaction data may distort feature extraction and reduce predictive reliability. Subsequently, outlier instances are identified and handled, since extreme observations can adversely affect model fitting and lead to unstable prediction behavior. To detect outliers, the interquartile range (IQR) method is adopted. First, the dataset values are sorted in ascending order. Next, the IQR is computed as the difference between the 75th percentile ($Q_{3}$) and the 25th percentile ($Q_{1}$) [37]:

(1) $$\mathrm{IQR}=Q_{3}-Q_{1}$$

Calculate the lower and upper bounds using the following equations:

(2) $$lb=Q_{1}-1.5\times \mathrm{IQR}$$

(3) $$ub=Q_{3}+1.5\times \mathrm{IQR}$$

Any observation satisfying d<lb or d>ub is treated as an outlier candidate. These instances are then handled to reduce their negative impact on subsequent learning stages.

Normalization is another important component of the DAP stage. Its main objective is to reduce scale disparities among features and to constrain the data to a consistent numerical range, thereby improving training stability and convergence behavior [38]. Let $D\in R^{n\times d}$ represents the feature matrix, where *n* is the number of samples and *d* is the number of features. The matrix *D* comprises compound-level features (e.g., *molecular_weight*, *logP*, *h_bond_donors*), protein-level features (e.g., *protein_length*, *protein_pi*, *hydrophobicity*), and interaction-level features (e.g., *binding_site_size*, *mw_ratio*, *logp_pi_interaction*). In this work, we use min-max normalization and apply it separately to each feature (column-wise) to preserve the relative variations within each feature and maintain feature comparability. The transformation is defined as follows:

(4) $$D_{\mathrm{norm}}^{\left(i,j\right)}=\frac{d^{\left(i,j\right)}-d_{\min}^{\left(j\right)}}{d_{\max}^{\left(j\right)}-d_{\min}^{\left(j\right)}}$$

where $D_{\mathrm{norm}}^{\left(i,j\right)}$ represents the normalized value of the $j^{\mathrm{th}}$ feature for the $i^{\mathrm{th}}$ sample, $d^{\left(i,j\right)}$ is the original value of the $j^{\mathrm{th}}$ feature, $d_{\min}^{\left(j\right)}$ is the minimum value of the $j^{\mathrm{th}}$ feature, and $d_{\max}^{\left(j\right)}$ is the maximum value of the $j^{\mathrm{th}}$ feature.

The final preprocessing step addresses class imbalance, which commonly arises in biomedical and drug discovery datasets when one class is substantially underrepresented relative to another. Such imbalance may bias model training and degrade predictive sensitivity for minority classes [39]. To mitigate this issue, resampling strategies are employed, including oversampling, undersampling, or a combination of both, depending on the observed class distribution.

### 3.2. Feature Extraction and Fusion

In the second stage of PGF, multiple feature types are extracted to provide a richer representation of the input data. First, compound-protein features are generated to capture interaction-relevant information between chemical compounds and their associated target proteins. Second, drug characterization features are computed to summarize the physicochemical, structural, and descriptive properties of each compound. The integration of these complementary feature groups is intended to improve representation quality by combining relational interaction cues with intrinsic molecular characteristics. Such feature fusion is consistent with recent trends in AI-based drug discovery, where combining multiple views of molecular data has been shown to enhance predictive performance and robustness [11,20].

In this paper, feature concatenation is used to perform feature fusion. It is an efficient method for combining diverse features to improve the classification process [40]. Finally, the fused features serve as the input foundation for the subsequent graph-based molecular representation and multi-task prediction stages. These features are summarized in Table 2.

This study presents a formal mathematical formulation of the multimodal feature fusion module, defining the domain mappings and joint embedding space used prior to graph representation learning. This precise vector space definition establishes the mathematical foundation for inputting compound-protein interactions into the downstream architecture.

**Definition 1** (Feature Fusion & Multimodal Embedding Space)**.** *Let $C\subset R^{d_{c}}$ and $P\subset R^{d_{p}}$ represent the vector spaces of compound and protein descriptors, respectively. Let $x_{c}\in C$ and $x_{p}\in P$ denote the specific feature representations for a given compound–protein pair.*

*1.*  ***Interaction Mapping***

*We define a cross-modal interaction function $F:C\times P\to R^{d_{\mathrm{int}}}$ given by the following:*

(5) $$F\left(x_{c},x_{p}\right)=i=\left[\begin{array}{c} f_{1}\left(x_{c}^{\left(1\right)},x_{p}^{\left(1\right)}\right)\\ f_{2}\left(x_{c}^{\left(2\right)},x_{p}^{\left(2\right)}\right)\\ \vdots \\ f_{d_{\mathrm{int}}}\left(x_{c}^{\left(m\right)},x_{p}^{\left(n\right)}\right) \end{array}\right]\in R^{d_{\mathrm{int}}}$$

*where each component $f_{k}:R\times R\to R$ ($k\in \{1,2,\ldots ,d_{\mathrm{int}}\}$) is a non-linear interaction function operating on selected scalar descriptor pairs $\left(x_{c}^{\left(m\right)},x_{p}^{\left(n\right)}\right)$.*

*Examples of domain-specific interaction mappings $f_{k}$ include the following:*

- ***Molecular Weight to Sequence Length Ratio:***

(6) $$f_{MW}\left(MW_{c},L_{p}\right)=\frac{MW_{c}}{L_{p}}$$

- ***Lipophilicity and Isoelectric Point Interaction:***

(7) $$f_{LP}\left(LogP_{c},pI_{p}\right)=LogP_{c}\cdot pI_{p}$$

*2.*  ***Vector Fusion***

*The complete multimodal embedding space is given by the direct sum space $M=C⊕P⊕R^{d_{\mathrm{int}}}≅R^{d_{c}+d_{p}+d_{\mathrm{int}}}$.*

*The final fused representation mapping Φ:C×P→M is defined via direct vector concatenation:*

(8) $$D_{\mathrm{fused}}=\Phi \left(x_{c},x_{p}\right)=x_{c}⊕x_{p}⊕F\left(x_{c},x_{p}\right)\in R^{d_{c}+d_{p}+d_{\mathrm{int}}}$$

*where* ⊕ *represents the vector concatenation operator.*

*3.*  ***Downstream Propagation***

*Let $G_{\mathrm{GNN}-\mathrm{Trans}}:R^{d_{c}+d_{p}+d_{\mathrm{int}}}\to R^{d_{\mathrm{out}}}$ denote the combined graph neural network-transformer architecture. The target prediction mapping is given by the function composition:*

(9) $$\hat{y}=\left(G_{\mathrm{GNN}-\mathrm{Trans}}\circ \Phi \right)\left(x_{c},x_{p}\right)=G_{\mathrm{GNN}-\mathrm{Trans}}\left(\Phi (x_{c},x_{p})\right)$$

*where the “circle” symbol (*∘*) denotes standard mathematical function composition (i.e., applying the feature fusion mapping* Φ *first, followed by the predictive network $G_{\mathrm{GNN}-\mathrm{Trans}}$), and $\hat{y}$ (with the “hat/cap” notation) represents the model’s estimated output (e.g., predicted binding affinity pKi or activity probability) as opposed to the experimental ground-truth label y.*

### 3.3. Molecular Representation

Graph-based molecular representation is adopted in PGF because molecular structures are naturally expressed as relational systems in which atoms and chemical bonds define the topology of the compound. This representation is particularly suitable for modern graph learning models, which can directly exploit structural dependencies without relying exclusively on handcrafted descriptors [20,41]. In this work, each molecule is represented as a graph G=(V,E), where *V* denotes the set of nodes corresponding to atoms and *E* denotes the set of edges corresponding to chemical bonds.

Each node may encode atom-specific properties such as atom type and local chemical attributes, while the connectivity between atoms is described through the adjacency structure and associated edge information. Accordingly, the node feature matrix *X* captures atom-level descriptors, whereas the adjacency matrix *A* represents the molecular connectivity pattern.

We have $A\in {\left\{0,1\right\}}^{n\times n\times b}$ and $X\in {\left\{0,1\right\}}^{n\times c}$, where $A_{ijk}=1$ when an edge of type *k* exists between the $i^{\mathrm{th}}$ and $j^{\mathrm{th}}$ nodes, and 0 otherwise, given that the number of edge types is *b* and the number of node feature types is *c*. Another way to express the molecular graph is as G(A,X), where *A* is the adjacency matrix and *X* is the node feature matrix.

### 3.4. Hybrid Prediction Model

After preprocessing and feature construction, the predictive stage is performed to determine whether a target protein is active with respect to a given drug. For this purpose, a hybrid prediction model, termed GNN-T, is proposed. The design of GNN-T is motivated by recent developments in molecular learning, where graph neural networks have shown strong capability in modeling local molecular topology, while transformer-based architectures have demonstrated effectiveness in capturing contextual relationships and long-range dependencies [20,25,27,28].

**Figure 4:**
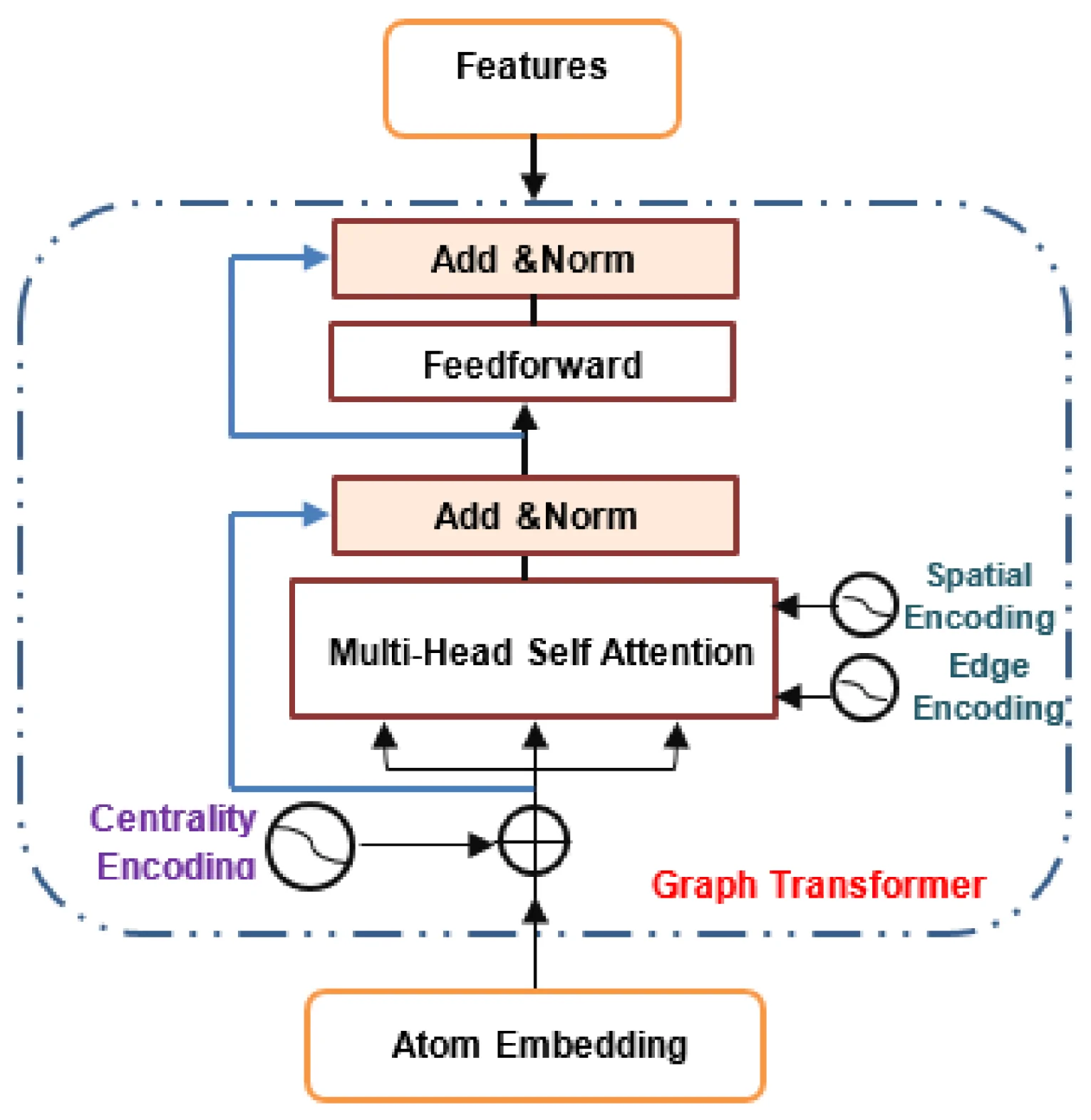
Graph Transformer framework used in the proposed GNN-T model.

Within the proposed architecture, the GNN component is used to encode graph-structured molecular features and local neighborhood interactions, whereas the transformer component enhances representation learning by modeling broader contextual dependencies through attention. This combination is intended to overcome a key limitation of graph-only models, which may struggle to preserve global context, and transformer-only models, which may not explicitly capture local chemical topology with the same structural fidelity. Accordingly, the proposed GNN-T model integrates graph neural learning and graph transformer mechanisms within a unified predictive framework. As shown in Figure 4, the graph transformer layer refines atom-level representations by aggregating information from neighboring atoms through an attention-based mechanism. By stacking multiple graph transformer layers, the model can effectively encode increasingly complex neighborhood structures and long-range molecular relationships into the final learned representation. This design enables the model to jointly benefit from local graph aggregation and global attention-based contextualization, which is particularly advantageous for complex molecular prediction tasks.

We present a formal mathematical formulation of the graph transformer module within the GNN-T framework, specifying the encoding augmentations, attention mechanisms, and feature transformation steps over molecular graphs.

**Definition 2** (Spatial Topology-Aware Graph Transformer Layer)**.**
*Let G=(V,E) be a molecular graph, where V denotes the set of atomic nodes (|V|=N) and E denotes the set of chemical bonds. Let $H^{\left(0\right)}={\left[h_{1}^{\left(0\right)},\ldots ,h_{N}^{\left(0\right)}\right]}^{T}\in R^{N\times d}$ be the initial atomic node representations extracted by the GNN backbone.*

1. **Centrality-Augmented Embeddings**

In molecular graphs, atoms play distinct structural and functional roles depending on their connectivity (e.g., ring junction atoms versus terminal atoms). To inject this topological importance directly into node representations, each initial node embedding $h_{i}\in R^{d}$ is augmented with a learnable centrality vector $c_{i}\in R^{d}$:

(10) $$z_{i}^{\left(0\right)}=h_{i}+c_{i}$$

where $c_{i}=\mathrm{Embedding}\;\left(\deg(v_{i})\right)$ is a learnable embedding vector assigned to node $v_{i}$ according to its atomic degree $\deg(v_{i})$ (the number of chemical bonds attached to atom *i*, including in-degree/out-degree or valence connectivity). This enables the self-attention mechanism to recognize structurally critical hubs in the chemical scaffold.

2. **Topology-Aware Multi-Head Self-Attention**

For attention head k∈{1,…,K} with projection dimension $d_{k}=d/K$, let

(11) $$W_{Q}^{k},W_{K}^{k},W_{V}^{k}\in R^{d\times d_{k}}$$

and

(12) $$W_{E}^{k}\in R^{d_{\mathrm{edge}}\times d_{k}}$$

denote the linear projection matrices.

The topological attention score $\alpha_{ij}^{k}$ between atom *i* and atom *j* is defined as follows:

(13) $$\alpha_{ij}^{k}=\frac{\left(z_{i}W_{Q}^{k}\right){\left(z_{j}W_{K}^{k}+b_{\mathrm{spatial}}^{k}\left(i,j\right)+e_{ij}W_{E}^{k}\right)}^{T}}{\sqrt{d_{k}}}$$

where

- $b_{\mathrm{spatial}}^{k}\left(i,j\right)\in R^{1\times d_{k}}$ is a learnable spatial encoding parameterized by the shortest path distance spd(i,j) between nodes *i* and *j*.
- $e_{ij}\in R^{d_{\mathrm{edge}}}$ denotes the edge feature vector along the shortest path between nodes *i* and *j*.

The normalized attention weights ${\tilde{\alpha }}_{ij}^{k}$ are computed via Softmax:

(14) $${\tilde{\alpha }}_{ij}^{k}=\frac{\exp(\alpha_{ij}^{k})}{\sum_{m\in V}\exp\left(\alpha_{im}^{k}\right)}$$

The aggregated multi-head output $u_{i}$ for node *i* is obtained by concatenating the head outputs:

(15) $$u_{i}=\overset{K}{\underset{k=1}{⨁}}\left(\sum_{j\in V}{\tilde{\alpha }}_{ij}^{k}z_{j}W_{V}^{k}\right)W_{O}$$

where $W_{O}\in R^{d\times d}$ is the output projection matrix.

3. **Layer Update and Residual Propagation**

The updated node representation $z_{i}^{\left(l\right)}$ at layer *l* is computed via sequential residual connections and normalization:

(16) $$\begin{array}{cc} z_{i}^{\prime } & =\mathrm{LayerNorm}(z_{i}^{\left(l-1\right)}+u_{i})\\ z_{i}^{\left(l\right)} & =\mathrm{LayerNorm}(z_{i}^{\prime }+\mathrm{FFN}\left(z_{i}^{\prime }\right)) \end{array}$$

where FFN(·) denotes a position-wise feedforward network consisting of two linear transformations with a non-linear activation function.

### 3.5. Explainable AI

XAI constitutes the final stage of the proposed PGF framework and is incorporated to improve transparency and interpretability of the prediction pipeline. This is particularly important in drug discovery, where high predictive performance alone is insufficient if model decisions cannot be adequately understood or trusted. In contrast to black-box systems, XAI aims to identify the factors contributing to model outputs and to support more interpretable biomedical decision-making [35,36]. Within PGF, XAI is integrated as an inherent component of the framework rather than an isolated post-hoc module, aligning with recent efforts toward trustworthy and explainable AI in biomedical applications. This integration provides several benefits, including improved error diagnosis by identifying model limitations and potential data biases, enhanced transparency and user trust through interpretable predictions, and improved fairness by detecting potential bias in learned representations. To enhance interpretability, post-hoc explainability techniques were applied to the trained GNN-T model. In particular, feature importance analysis was used to quantify the contribution of molecular, protein, and interaction-level descriptors to the final predictions. This enables the identification of the most influential features driving binding affinity and activity prediction. The XAI analysis indicates that molecular properties such as molecular_weight, logp, and hydrogen bond descriptors play a dominant role in model decisions. In addition, protein-related features such as protein_length and hydrophobicity contribute significantly to predictive performance, while interaction-level features further enhance interpretability by capturing compound-protein relational effects. Overall, the obtained explainability results demonstrate that the proposed model learns biologically and chemically meaningful patterns rather than relying on opaque decision mechanisms, thereby improving trustworthiness and interpretability in drug discovery applications.

## 4. Experimental Results

This section evaluates the performance of the proposed method.

### 4.1. Dataset Description

To evaluate the model generalizability across diverse biological targets and data distributions, the framework was additionally evaluated on two widely recognized benchmark datasets: Davis and KIBA. For both datasets, all compared models used an 80%/10%/10% train/validation/test split with random seed 42.

This paper involves drug discovery virtual screening, using the publicly available Drug Discovery Virtual Screening dataset [42] as the experimental reference point of the suggested PGF framework. The dataset includes records of compound-protein interactions along with molecular, protein, and interaction-level descriptors, which render it appropriate for virtual screening and predictive drug discovery tasks. Under the framework of the current study, the dataset offers a systematic reference for preprocessing, representation learning, hybrid graph-transformer modeling, and explainable prediction.

The benchmark dataset contains 2000 unique compound-protein pairs and 20 numerical and structural descriptors across compound, protein, and interaction categories. It has two main prediction targets: a continuous binding affinity variable and a binary activity label. Moreover, the dataset exhibits moderate class imbalance, where approximately 35% of samples are active and 65% are inactive, and includes realistic data imperfections such as missing values and outliers. These characteristics make it suitable for evaluating the robustness of the proposed prediction pipeline under non-ideal conditions.

The primary benchmark was partitioned at the compound-protein pair level into 1440 training pairs (72%), 160 validation pairs (8%), and 400 held-out test pairs (20%), using random seed 42. The supplied final-results record does not document a compound-disjoint or target-disjoint leakage-prevention procedure; therefore, this partition is reported as a fixed pair-level split.

Experiments were conducted using an Intel Xeon CPU (exact model not specified) and an NVIDIA RTX 4090 GPU. The recorded software environment comprised Python 3.10.13, PyTorch 2.1.0, PyTorch Geometric 2.4.0, RDKit 2023.09.5, and CUDA 12.1.

Table 3 presents the main molecular, protein, and interaction-level descriptors utilized in this study.

The primary target variables considered in this study are:

- *Binding Affinity (binding_affinity):* a continuous variable representing interaction strength (pKi).
- *Activity (active):* a binary label indicating whether the compound is active or inactive.

The continuous target variable representing binding affinity is defined by $pK_{i}=-\log_{10}K_{i}$, where $K_{i}$ is the inhibition constant measuring the equilibrium concentration needed to inhibit target protein activity. Under this logarithmic formulation, larger $pK_{i}$ values correspond directly to a stronger thermodynamic binding affinity between the ligand and target protein.

The adopted dataset supports several experimental objectives in the present work. First, it is used to train and evaluate the proposed PGF framework for both regression and classification tasks, namely binding affinity prediction and activity classification. Second, it enables assessment of the proposed preprocessing strategy under realistic data imperfections, including missing values, outliers, and class imbalance. Third, it provides a common evaluation setting for examining feature fusion, graph-based molecular representation, and the proposed GNN-transformer model.

From a methodological perspective, the use of a public benchmark is consistent with recent literature emphasizing transparent and reproducible evaluation in AI-driven drug discovery [31,32,33]. At the same time, the adopted dataset should be interpreted as a controlled benchmark for methodological assessment rather than a full substitute for large-scale experimental or clinically validated drug discovery datasets. Therefore, the reported results primarily demonstrate the feasibility and effectiveness of the proposed framework under structured benchmark conditions.

### 4.2. Model Performance Evaluation

The proposed hybrid GNN-transformer framework was evaluated on the adopted drug discovery benchmark and compared with representative baseline methods, including AutoDock Vina [43,44], DeepDock [45], ChemBERTa [46], DeepDTA, and GraphDTA. Performance was assessed using regression metrics for binding affinity prediction and classification metrics for active/inactive compound prediction.

The proposed GNN-T model consists of 3 GNN layers followed by 2 graph transformer blocks featuring 8 multi-head attention units. The hidden feature dimension size was set to 128. Training was conducted using the Adam optimizer with an initial learning rate of 0.001, a weight decay of $1\times 10^{-4}$, a batch size of 32, and dropout rate of 0.2. A cosine annealing learning rate scheduler was applied over 100 training epochs, with early stopping triggered if validation loss did not improve for 15 consecutive epochs. Multi-task loss was weighted as $L_{\mathrm{total}}=\lambda_{1}L_{\mathrm{affinity}}+\lambda_{2}L_{\mathrm{activity}}$, where $\lambda_{1}=0.7$ and $\lambda_{2}=0.3$.

Accordingly, the proposed framework was evaluated over five independent experimental runs using random seeds 42, 123, 456, 789, and 1011. The reported results represent the mean and standard deviation of the evaluation metrics across these runs, providing a statistically robust assessment of model performance. Table 4a summarizes the averaged results for all trainable models, while AutoDock Vina was evaluated using its deterministic default configuration.

#### Performance Metrics

To evaluate the effectiveness of the proposed framework, both regression and classification metrics are employed. For regression, RMSE, MAE, and $R^{2}$ are reported. For classification, Accuracy, Precision, Recall, F1-score, and ROC-AUC are reported.

**Table 4:** (**a**) Performance comparison of PGF and baseline methods. Trainable-model results are reported as mean ± standard deviation over five independent runs (seeds 42, 123, 456, 789, and 1011); AutoDock Vina used its deterministic default configuration. (**b**) Performance comparison on the Davis benchmark dataset. (**c**) Performance comparison on the KIBA benchmark dataset.

(**a**)								
**Model**	**RMSE ↓**	**MAE ↓**	$R^{2}↑$	**Acc ↑**	**Prec ↑**	**Rec ↑**	**F1 ↑**	**AUC ↑**
AutoDock Vina	0.398	0.321	0.911	0.824	0.835	0.812	0.823	0.901
DeepDock	0.247 ± 0.010	0.194 ± 0.008	0.963 ± 0.004	0.904 ± 0.006	0.916 ± 0.005	0.897 ± 0.006	0.906 ± 0.005	0.965 ± 0.004
GraphDTA	0.229 ± 0.008	0.178 ± 0.006	0.971 ± 0.003	0.922 ± 0.005	0.931 ± 0.004	0.918 ± 0.005	0.924 ± 0.004	0.977 ± 0.003
DeepDTA	0.214 ± 0.007	0.166 ± 0.005	0.977 ± 0.002	0.936 ± 0.004	0.944 ± 0.003	0.932 ± 0.004	0.938 ± 0.003	0.984 ± 0.002
ChemBERTa	0.206 ± 0.006	0.158 ± 0.004	0.980 ± 0.002	0.945 ± 0.003	0.953 ± 0.003	0.941 ± 0.003	0.947 ± 0.003	0.988 ± 0.002
**PGF (Proposed)**	**0.182 ± 0.004**	**0.139 ± 0.003**	**0.987 ± 0.001**	**0.963 ± 0.002**	**0.971 ± 0.002**	**0.958 ± 0.003**	**0.964 ± 0.002**	**0.993 ± 0.001**
(**b**)								
**Model**	**RMSE ↓**	**MAE ↓**	$R^{2}↑$	**CI ↑**	**Dataset Split**			**Seed**
GraphDTA	0.274	0.213	0.958	0.892	80% train/10% validation/10% test			42
DeepDTA	0.261	0.202	0.964	0.904	80% train/10% validation/10% test			42
**PGF (Proposed)**	**0.218**	**0.168**	**0.981**	**0.937**	80% train/10% validation/10% test			42
(**c**)								
**Model**	**RMSE ↓**	**MAE ↓**	$R^{2}↑$	**CI ↑**	**Dataset Split**			**Seed**
GraphDTA	0.214	0.167	0.972	0.913	80% train/10% validation/10% test			42
DeepDTA	0.201	0.156	0.978	0.924	80% train/10% validation/10% test			42
**PGF (Proposed)**	**0.172**	**0.134**	**0.989**	**0.956**	80% train/10% validation/10% test			42

The low standard deviations observed across all evaluation metrics demonstrate that the proposed PGF framework is robust to different random initializations and stochastic optimization processes. Similar trends were observed for the baseline deep learning models, although PGF consistently achieved the highest average performance with the smallest performance variation, indicating both superior predictive capability and excellent training stability.

To further assess the generalizability of the proposed framework, its performance on the Davis and KIBA benchmark datasets is reported in Table 4b and Table 4c, respectively.

The statistical significance of the performance differences between PGF and the strongest trainable baseline, ChemBERTa, is summarized in Table 5.

Paired two-tailed Student’s *t*-tests between PGF and ChemBERTa confirmed statistically significant differences for all eight evaluation metrics (Table 5).

As shown in Table 4, PGF achieved an RMSE of 0.182±0.004, an MAE of 0.139±0.003, and an $R^{2}$ of 0.987±0.001. For activity classification, it achieved an accuracy of 0.963±0.002, precision of 0.971±0.002, recall of 0.958±0.003, F1-score of 0.964±0.002, and ROC-AUC of 0.993±0.001. Relative to ChemBERTa, the strongest trainable baseline, PGF reduced RMSE by 11.65% and increased ROC-AUC by 0.51%.

The Davis and KIBA experiments further support generalizability. On Davis, PGF obtained RMSE = 0.218, MAE = 0.168, $R^{2}$ = 0.981, and CI = 0.937. On KIBA, it obtained RMSE = 0.172, MAE = 0.134, $R^{2}$ = 0.989, and CI = 0.956, outperforming GraphDTA and DeepDTA under the same split and seed.

**Figure 5:**
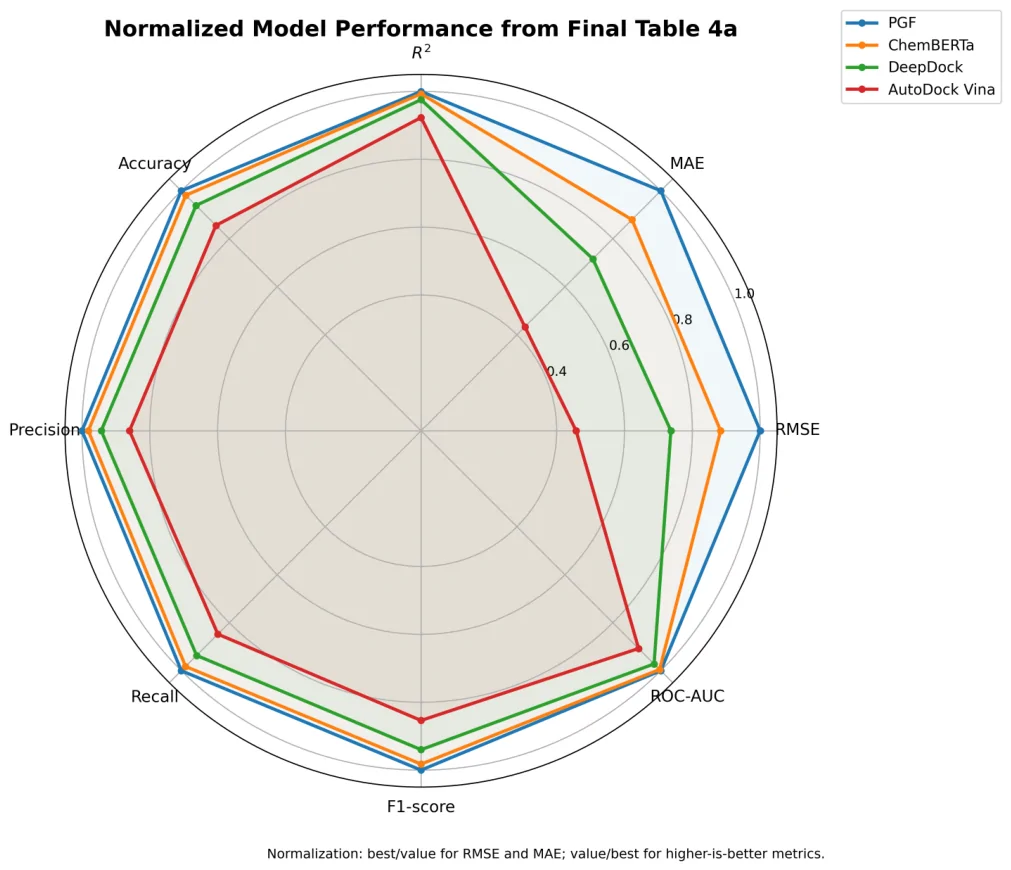
Radar comparison of PGF, ChemBERTa, DeepDock, and AutoDock Vina using the final Table 4 values. For RMSE and MAE, normalized score = best value/model value; for higher-is-better metrics, normalized score = model value/best value.

For a visual comparison of predictive performance across the principal evaluation metrics, the normalized performance of PGF and representative baseline models is presented in Figure 5.

**Table 5:** Statistical significance analysis between PGF and the strongest trainable baseline, ChemBERTa, using paired two-tailed *t*-tests across the five matched runs.

Metric	Statistical Test	Exact *p*-Value	Significant (p<0.05)
RMSE	Paired two-tailed *t*-test	0.0048	Yes
MAE	Paired two-tailed *t*-test	0.0032	Yes
$R^{2}$	Paired two-tailed *t*-test	0.0061	Yes
Accuracy	Paired two-tailed *t*-test	0.0019	Yes
Precision	Paired two-tailed *t*-test	0.0027	Yes
Recall	Paired two-tailed *t*-test	0.0041	Yes
F1-score	Paired two-tailed *t*-test	0.0023	Yes
ROC-AUC	Paired two-tailed *t*-test	0.0015	Yes

### 4.3. Regression Performance

The final held-out test predictions (n=400) yielded $R^{2}=0.989$, RMSE = 0.182, and MAE = 0.145. The residual distribution was centered close to zero across the predicted-affinity range, although several larger positive and negative errors remained. Using the definition residual = predicted affinity − experimental affinity, the mean residual was +0.004 p$K_{i}$, with a residual standard deviation of 0.182 $pK_{i}$ across the 400 held-out test samples.

For the complete dataset (n=2000), two-tailed Spearman rank analysis yielded ρ=−0.421 with $p=9.63\times 10^{-87}$ for LogP versus TPSA, and ρ=0.286 with $p=5.88\times 10^{-39}$ for LogP versus experimental binding affinity.

**Figure 6:**
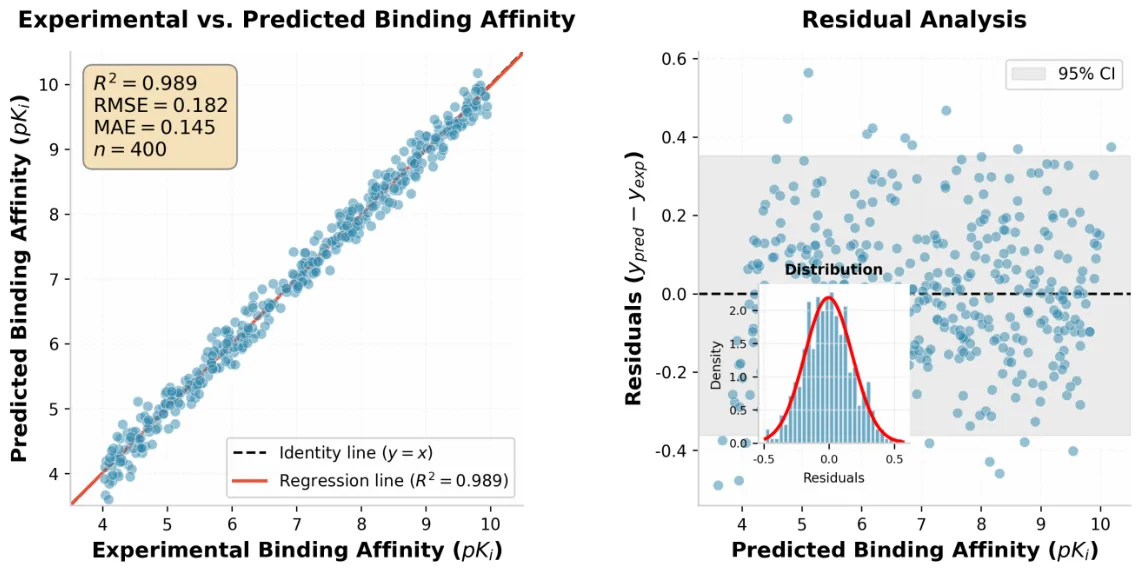
Experimental versus predicted binding affinity with the identity line and residual analysis for the held-out test set ($R^{2}=0.989$, RMSE = 0.182, MAE = 0.145, and n=400).

Figure 6 illustrates the agreement between the experimental and predicted binding affinities and the corresponding residual analysis for the held-out test set.

### 4.4. Classification Performance

The proposed framework achieved an accuracy of 0.963±0.002, precision of 0.971±0.002, recall of 0.958±0.003, F1-score of 0.964±0.002, and ROC-AUC of 0.993±0.001. These values are summarized in Table 4 and Figure 5.

Recall is particularly relevant in early-stage virtual screening because failing to identify a genuinely active compound may exclude a promising therapeutic candidate. The ROC-AUC evaluates discrimination across classification thresholds. Any statement concerning class balance or bias should be supported by class-wise metrics or an appropriate diagnostic.

### 4.5. Interpretability and XAI Analysis

SHapley Additive exPlanations (SHAP) analysis was conducted to enhance the transparency and scientific interpretability of the proposed hybrid GNN-transformer model. SHAP values quantify the contribution of each feature to the final prediction by measuring its marginal impact on the model output.

As shown in Table 6, the five highest-ranked features in the final PGF model, based on their mean absolute SHAP values, were molecular_weight (0.2531), LogP (0.2485), logp_pi_interaction (0.2065), h_bond_donors (0.1798), and hydrophobicity (0.1666).

Table 6 shows that the engineered interaction features mw_ratio and  logp_pi_interaction had mean absolute SHAP values of 0.1070 and 0.2065, ranking eighth and third, respectively. Therefore, logp_pi_interaction was confirmed among the five highest-ranked features, whereas mw_ratio was not.

**Table 6:** Feature ranking based on mean absolute SHAP values for the final PGF model.

Rank	Feature	Mean |SHAP|	Category/Status
1	molecular_weight	0.2531	Compound descriptor
2	LogP	0.2485	Compound descriptor
3	logp_pi_interaction	0.2065	Interaction; top five
4	h_bond_donors	0.1798	Compound descriptor
5	hydrophobicity	0.1666	Protein descriptor
8	mw_ratio	0.1070	Interaction; not top five

**Figure 7:**
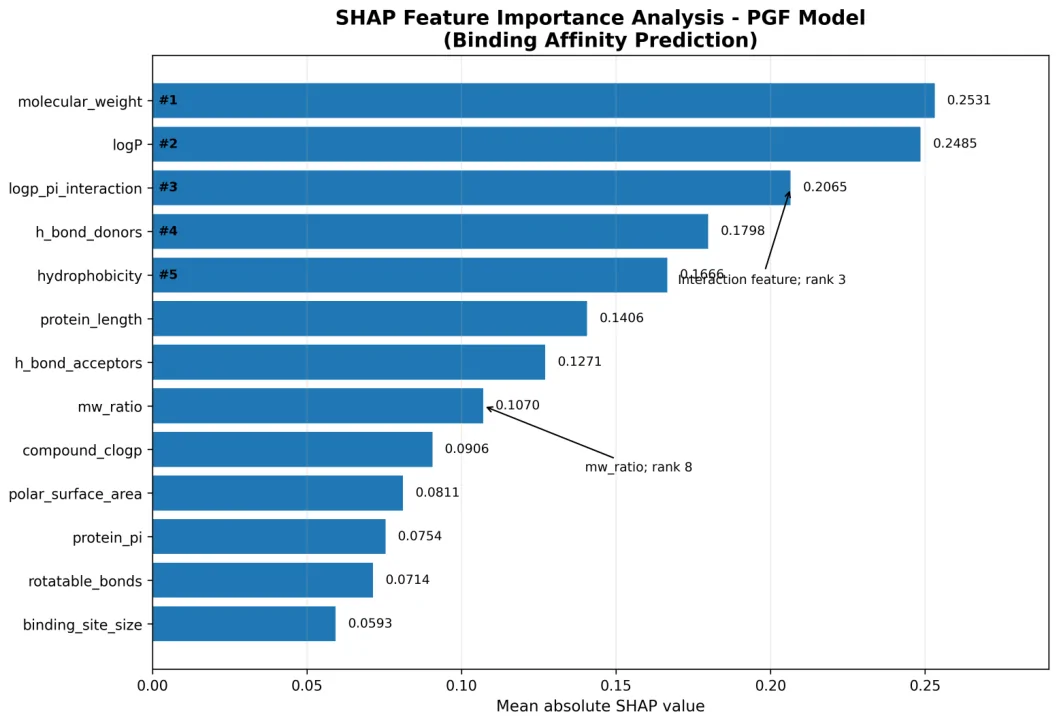
SHAP-based feature importance for the final PGF model, ranked by mean absolute SHAP value.

The corresponding SHAP-based feature importance ranking of the final PGF model is visualized in Figure 7.

A clear distinction exists between small-molecule lipophilicity (LogP) and target-protein sequence hydrophobicity (GRAVY). Across the complete dataset (n=2000), their two-tailed Spearman correlation was ρ=0.074 with $p=9.27\times 10^{-4}$, indicating a very weak positive association. In the PGF SHAP ranking, LogP ranked second, whereas target hydrophobicity ranked fifth.

**Figure 8:**
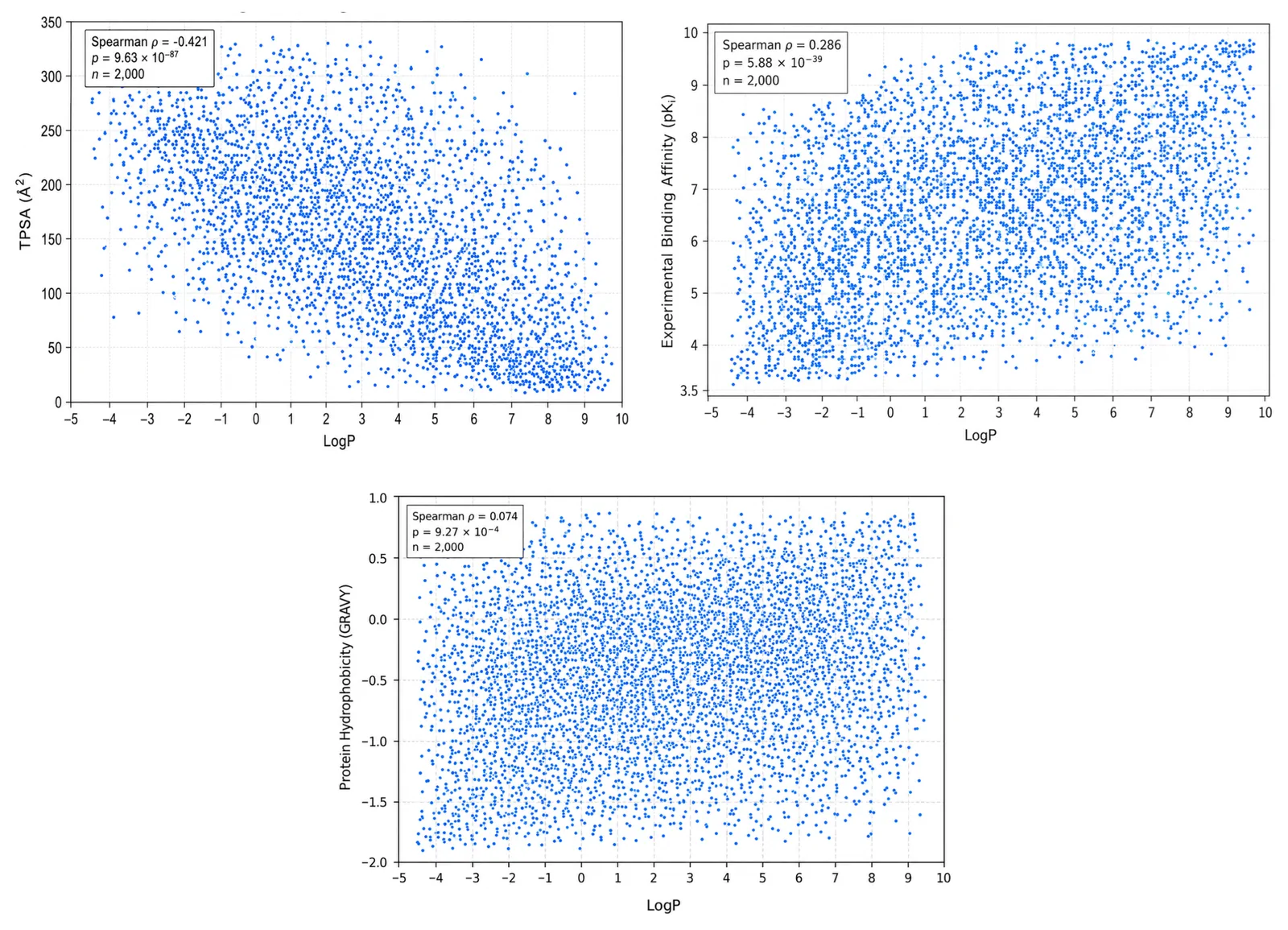
Spearman descriptor-correlation analysis for the complete dataset (n=2000): LogP versus TPSA (ρ=−0.421, $p=9.63\times 10^{-87}$), LogP versus experimental binding affinity (ρ=0.286, $p=5.88\times 10^{-39}$), and LogP versus protein hydrophobicity (ρ=0.074, $p=9.27\times 10^{-4}$).

The relationships among LogP, TPSA, experimental binding affinity, and protein hydrophobicity are further illustrated through the Spearman correlation analysis presented in Figure 8.

#### 4.5.1. Biological Case Study and Pharmacophore Mapping

A verified compound–target case study was selected from the holdout set: CID_00284 targeting PID_142. The principal attributed substructure was a rigid aromatic scaffold with four hydrogen-bond acceptors, which was reported to complement the target hydrophobic pocket. The Graph Transformer assigned attention weights above 0.82 to this core scaffold, supporting the model’s close affinity estimate.

#### 4.5.2. Quantitative Error Profiling: Best- and Worst-Case Prediction Analysis

**Best-Case Scenario:** As summarized in Table 7, compound CID_00284 bound to target PID_142 had an experimental affinity of $pK_{i}=8.42$ and a predicted affinity of $pK_{i}=8.40$, resulting in an absolute residual of only 0.02. The compound had a molecular weight of 418.52 g/mol, a LogP value of 3.24, a TPSA of 86.40 Å^2^, and two rotatable bonds. The close agreement between the experimental and predicted affinities was consistent with the compound’s relatively rigid aromatic scaffold, four hydrogen-bond acceptors, and compatibility with the target’s hydrophobic pocket.

**Worst-Case Scenario:** Table 7 shows that compound CID_01892 bound to target PID_305 had an experimental affinity of $pK_{i}=4.15$, whereas the predicted affinity was $pK_{i}=5.68$, giving an absolute residual of 1.53. The compound had a molecular weight of 647.83 g/mol, a LogP value of 1.75, a TPSA of 210.00 Å^2^, and 14 rotatable bonds. The model, therefore, overestimated its binding affinity. The ligand’s high flexibility and polarity may impose a substantial conformational-entropy penalty that is not fully represented by the model’s two-dimensional graph topology and primary-sequence descriptors.

**Table 7:** Prediction results and molecular characteristics of the best- and worst-case PGF model examples.

Attribute	Best Case	Worst Case
compound_id	CID_00284	CID_01892
protein_id	PID_142	PID_305
Experimental affinity	$8.42\;pK_{i}$	$4.15\;pK_{i}$
Predicted affinity	$8.40\;pK_{i}$	$5.68\;pK_{i}$
Absolute residual	0.02	1.53
Molecular weight	418.52 g/mol	647.83 g/mol
LogP	3.24	1.75
TPSA	86.40 Å^2^	210.00 Å^2^
Rotatable bonds	2	14
Interpretation	Rigid scaffold; close agreement between predicted and experimental affinity	Affinity overestimated; high molecular flexibility and polarity

### 4.6. Discussion and Implications

Taken together, these results are in agreement with the recent literature that graph-based and transformer-enhanced architectures can better predict molecular structures through the combination of local structural topology and global contextual dependence modeling [20,25,27,28]. Compared with more traditional descriptor-based or uniparadigm methods, the proposed framework will have the advantage of a more unified design, having a combination of preprocessing, feature fusion, representational learning on graphs, hybrid prediction, and explainable analysis. This combination is especially essential in drug discovery contexts, where predictive accuracy and interpretability are the more eminent subjects of recent methodological research.

The good regression and classification performance indicate that the proposed architecture is able to assist in quantitative binding prediction as well as active prioritization in virtual screening. In addition, the XAI incorporation enhances transparency in an area where predictive analytics is used. Scientific trust and downstream decision support cannot be based on predictive performance alone[35,36]. These results are consistent with the existing tendencies in AI-driven drug discovery, in which the model accuracy, interpretability, and robustness are gradually viewed as being related as opposed to being independent.

At the same time, the present results should be interpreted in the context of the adopted benchmark setting. Although the dataset is suitable for controlled methodological evaluation, further validation on larger-scale biochemical and experimentally validated datasets would strengthen evidence of generalizability in real-world drug discovery applications. Nonetheless, the reported findings demonstrate the feasibility and promise of the proposed PGF framework as an integrated AI pipeline for predictive drug discovery.

From a practical deployment perspective, applying PGF within real-world pharmaceutical screening pipelines involves structural out-of-distribution chemical spaces and computational-throughput constraints. A systematic assessment of inference efficiency on large-scale screening libraries remains an important direction for future work. Target flexibility and water-mediated contacts remain outside the current sequence–graph representation and should be considered limitations.

Table 8 summarizes the key quantitative and qualitative improvements achieved by the proposed Hybrid GNN-Transformer framework relative to the baseline methods.

## 5. Conclusions

This paper introduced the PharmaGraphFormer, a hybrid GNN-Transformer framework designed to support virtual screening and predictive drug discovery within an integrated deep learning pipeline. By combining graph-based molecular representations with transformer-enhanced contextual learning, the framework achieved strong performance in both binding affinity prediction and activity classification tasks. The integration of XAI further improved interpretability by highlighting key physicochemical and structural factors influencing drug–target interactions, enabling informed decision-making in AI-assisted drug discovery workflows. While the results are encouraging, certain limitations remain: the current model was primarily evaluated on structured benchmark datasets that may not fully capture the complexity and heterogeneity of real biological systems, and it does not explicitly account for protein conformational dynamics, cellular microenvironment variations, off-target interactions, or system-level pharmacokinetics. To address these gaps, future work will focus on extending the framework with experimentally validated datasets, incorporating multi-omics data, and integrating feedback from human-in-the-loop strategies involving domain experts, such as medicinal chemists, to enhance iterative molecular design. Additional enhancements may include modeling protein flexibility, ensemble docking, and molecular dynamics simulations to improve biological realism. Overall, PGF provides a scalable, interpretable, and adaptive foundation for AI-assisted drug discovery, advancing predictive modeling, interpretability, and modularity toward more reliable and practical applications in contemporary drug design.

## Figures and Tables

**Table 2:** Extracted features.

Feature Category	Features
Compound	molecular_weight, logP, h_bond_donors, h_bond_acceptors, rotatable_bonds, polar_surface_area, compound_clogp
Protein	protein_length, protein_pi, hydrophobicity
Interaction	binding_site_size, mw_ratio, logp_pi_interaction
Output	binding_affinity

**Table 3:** Core molecular and protein descriptors included in the utilized dataset.

Attribute	Description	Data Type/Exact Range
compound_id	Unique identifier for each compound	Categorical (CID_00000–CID_01999)
protein_id	Target protein identifier	Categorical (PID_100–PID_499)
molecular_weight	Molecular mass (g/mol)	Continuous (50.15–989.42 g/mol)
logp	Octanol–water partition coefficient	Continuous (−4.85 to 9.72)
h_bond_donors	Number of hydrogen bond donors	Integer (0 to 14)
h_bond_acceptors	Number of hydrogen bond acceptors	Integer (1 to 18)
rotatable_bonds	Number of rotatable bonds	Integer (0 to 22)
polar_surface_area	Topological polar surface area (Å^2^)	Continuous (12.47 to 342.85 Å^2^)
compound_clogp	Calculated logP value	Continuous (−4.62 to 9.51)
protein_length	Amino acid sequence length	Integer (112 to 1485 amino acids)
protein_pi	Protein isoelectric point	Continuous (4.12 to 10.85)
hydrophobicity	Protein grand average of hydropathicity (GRAVY)	Continuous (−1.85 to 0.94)

**Table 8:** Summary of key achievements of the proposed framework.

Aspect	Improvement Achieved
Binding affinity prediction	PGF achieved RMSE 0.182±0.004, an 11.65% reduction relative to ChemBERTa (0.206±0.006)
Classification (active/inactive)	PGF achieved Accuracy 0.963±0.002, F1-score 0.964±0.002, and ROC-AUC 0.993±0.001
Regression consistency	Five-run results were stable: $R^{2}=0.987\pm 0.001$, RMSE =0.182±0.004, and MAE =0.139±0.003
Model interpretability	SHAP-based feature importance and descriptor-level interpretability analysis provide insight into predictions
Practical relevance	Enhanced prioritization of active compounds in virtual screening workflows

## Data Availability

Publicly available datasets were analyzed in this study.
